# Motion correction in optoacoustic mesoscopy

**DOI:** 10.1038/s41598-017-11277-y

**Published:** 2017-09-04

**Authors:** Mathias Schwarz, Natalie Garzorz-Stark, Kilian Eyerich, Juan Aguirre, Vasilis Ntziachristos

**Affiliations:** 1 Chair of Biological Imaging, Technical University of Munich, Ismaningerstr. 22, 81675 Munich, Germany; 2 Institute of Biological and Medical Imaging, Helmholtz Zentrum München, German Research Center for Environmental Health (GmbH), Ingolstädter Landstr. 1, 85764 Neuherberg, Germany; 3 iThera Medical GmbH, Zielstattstraße 13, 81379 Munich, Germany; 4 Department of dermatology and allergy, Technical University of Munich and ZAUM – Center of allergy and environment, HMGU and Technical University of Munich, Munich, Germany

## Abstract

Raster-scan optoacoustic mesoscopy (RSOM), also termed photoacoustic mesoscopy, offers novel insights into vascular morphology and pathophysiological biomarkers of skin inflammation *in vivo* at depths unattainable by other optical imaging methods. Using ultra-wideband detection and focused ultrasound transducers, RSOM can achieve axial resolution of 4 micron and lateral resolution of 20 micron to depths of several millimeters. However, motion effects may deteriorate performance and reduce the effective resolution. To provide high-quality optoacoustic images in clinical measurements, we developed a motion correction algorithm for RSOM. The algorithm is based on observing disruptions of the ultrasound wave front generated by the vertical movement of the melanin layer at the skin surface. From the disrupted skin surface, a smooth synthetic surface is generated, and the offset between the two surfaces is used to correct for the relative position of the ultrasound detector. We test the algorithm in measurements of healthy and psoriatic human skin and achieve effective resolution up to 5-fold higher than before correction. We discuss the performance of the correction algorithm and its implications in the context of multispectral mesoscopy.

## Introduction

Raster-scan optoacoustic (photoacoustic) mesoscopy (RSOM) is a biomedical optical imaging technique^1, 2^, capable of visualizing tissue morphology^3, 4^ and pathophysiological biomarkers of inflammatory diseases^5^ through several millimeters of depth while preserving high resolution. Exploiting ultra-wideband (UWB) ultrasound detectors the technique has recently demonstrated label free imaging and quantification of inflammatory biomarkers in psoriasis and eczema^5^. Moreover, multi-wavelength illumination and spectral unmixing allows visualization of a larger number of pathophysiological features, including tissue/blood oxygenation, melanin patterns^6^, or the bio-distribution of externally administered photo-absorbing labels^7^.

RSOM image formation is achieved by raster-scanning a focused ultrasound detector over the region of interest (ROI) to collect optoacoustic waves generated in the tissue in response to pulsed laser illumination. The focal point of the ultrasound detector lies slightly above the surface of the sample, and the detector collects ultrasound signals over an acceptance angle (aperture) defined by the area and focusing characteristics of the detecting element. The collected data are tomographically reconstructed to yield an image of the light absorbers within the skin. Data acquisition speed is determined by the pulse repetition rate of the laser, which cannot exceed the limits imposed by laser safety regulations^8, 9^. Generating a two-dimensional skin scan based on a grid of 250 × 250 points may require ~60 seconds when the laser has a pulse repetition rate (PRR) of 1 kHz; this scan time is longer when larger grids, multi-wavelength illumination, lower laser safety limits or data averaging are used^1, 3–6, 8, 10–14^. During this time, the subject’s movement, e.g. breathing motion at a respiratory rate of up to 30 times per minute^15, 16^, can alter the position of the ultrasound detector relative to the sample, affecting RSOM image quality^8, 17^. Restricting human motion during RSOM scanning is challenging. The RSOM scan head, which is connected to the main RSOM console by a semi-rigid arm, is usually attached to the ROI using suction or adhesive tape restricting lateral movement of the ROI. Nevertheless, motion effects are not entirely eliminated since the skin surface can possibly move relative to the acoustic axis of the detector due to possible deformation of the soft coupling media, such as water, acoustic gel, and thin, acoustically transparent membranes^3, 4, 10, 17–22^.

It is therefore important to explore image processing methods that can reduce the effects of such motion on RSOM imaging performance. We have previously developed an algorithm to correct for macroscopic motion in small animals. In this approach, mouse thorax cross-sections are clustered based on the cardiac cycle^23^. Clustering has also been employed to correct for motion artifacts due to the breathing cycle^24^. However, these clustering approaches are used to classify inter-frame motion of image frames, where each frame (image) is based on a reconstruction following a single laser pulse. RSOM, in contrast, involves tomographic reconstruction based on a set of recorded time signals and requires a motion correction approach targeting inner-frame motion.

In this work, we aimed to develop to our knowledge the first motion correction algorithm for RSOM. We hypothesize that by tracking the skin surface based on melanin content at each scan point, we could generate a synthetic surface, representative of skin motion. We further postulated that we could use this synthetic surface to correct for sample movement in the axial direction of the transducer during data acquisition, significantly reducing motion artifacts. To test this hypothesis, we developed a motion extraction and correction algorithm and measured a benign nevus with high-frequency RSOM (20–180 MHz). We interrogated the performance of the algorithm by studying motion artifact removal in single capillary loops, achieving effective resolution up to 5-fold better than before correction. We measured healthy and psoriatic skin with clinical RSOM (14–126 MHz), and demonstrate the correction of breathing motion, which allows for reliable imaging in a clinical context. Finally, we measured blood oxygenation and melanin content in healthy human skin with multispectral RSOM (14–126 MHz). We examined the performance of the algorithm by tracing the level of blood oxygenation along single vessels, and show that motion correction is necessary for correcting corrupted readings of blood oxygenation.

## Methods

### RSOM and the motion problem

**Figure 1:**
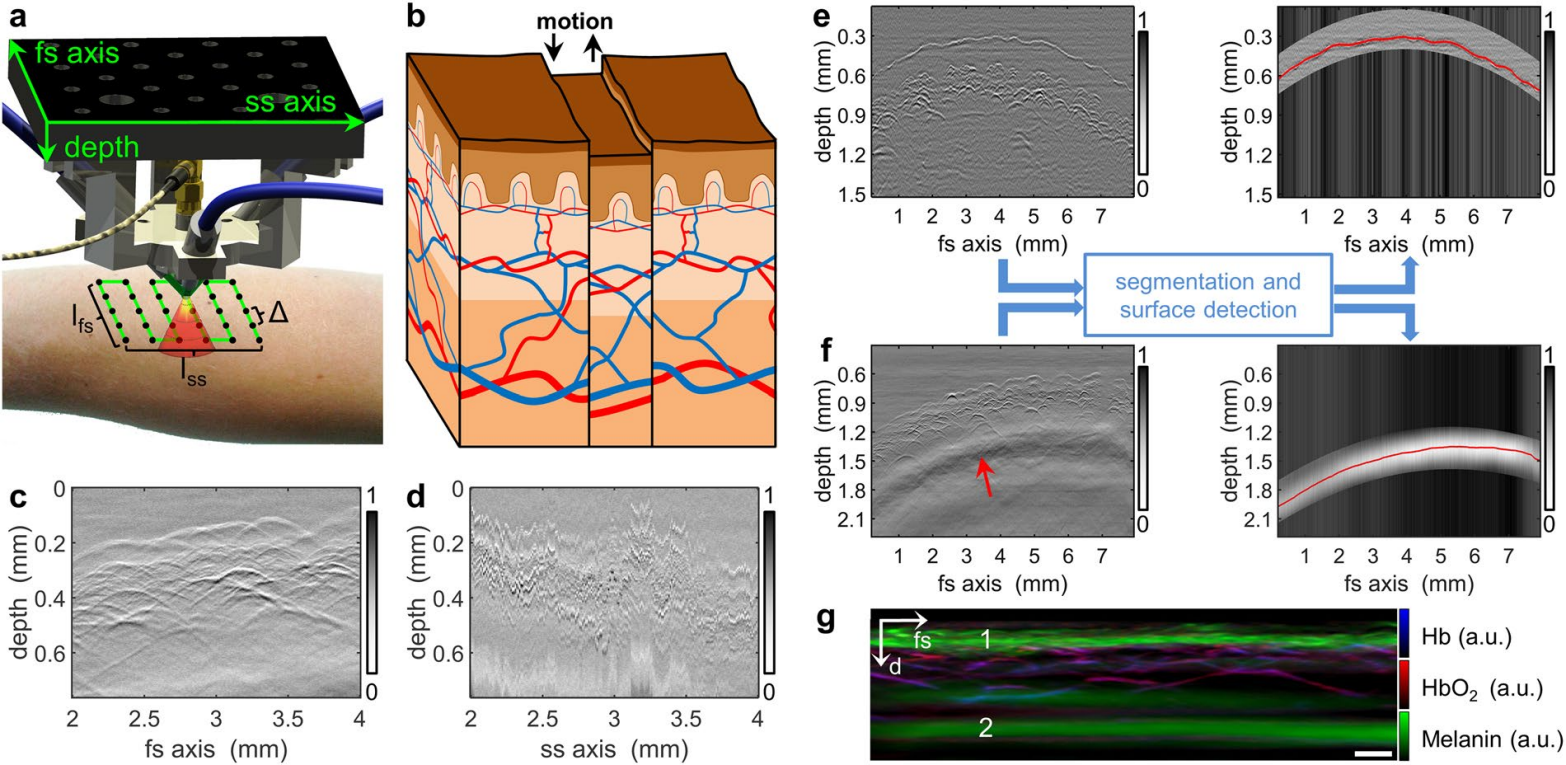
Principle of the proposed motion correction algorithm for RSOM. **(a**) Schematic of the detector-illumination unit, which is raster-scanned over the region of interest at a step size of Δ to give final dimensions l_fs_ × l_ss_. The detector features a cone-shaped sensitivity field (red/yellow). **(b)** Schematic illustrating how movement (black arrows) during RSOM scanning causes discontinuities in the surface of the epidermal layer as well as disruption of vessels. **(c)** B-scan of healthy human skin along the fs-axis, showing no discontinuities between subsequent positions. **(d)** B-scan of the sample in panel c along the ss-axis, showing large motion-induced artifacts. **(e)** B-scan of healthy human palm (left panel) after segmentation and surface detection were applied (right panel). The *stratum corneum* is separated clearly from the dermal papillae. (**f**) B-scan of healthy lower arm (left panel) with segmentation and surface detection applied (right panel). Segmentation was performed using only the low-frequency optoacoustic signal 600 µm below the skin surface (red arrow). The red curve indicates the maximum value of the low-frequency band. **(g)** Multispectral vertical MAP of healthy lower arm reconstructed without bandpass filtering. The low-frequency signal (*2*) shows the spectral signature of melanin and follows the shape of the epidermal layer (1). Scale bar, 250 µm. Abbreviations: d: depth; fs: fast-scanning; Hb, deoxyhemoglobin; HbO_2_, oxyhemoglobin; ss, slow-scanning.

RSOM involves raster-scanning a focused ultrasound detector over the ROI and recording the ultrasound waves generated in tissue in response to pulsed laser illumination (Fig. 1a). The collected data are tomographically reconstructed into an image using three-dimensional beam forming^1, 6, 14^. Typically, the ultrasound detector is scanned along a fast-scanning axis (fs-axis) and a slow-scanning axis (ss-axis) (Fig. 1a). One-dimensional time signals (making up the so-called “A-scan”) are recorded at each scan point, collecting ultrasound signal generated within the cone-shaped sensitivity field of the focused detector. One-dimensional scanning of the detector along the fs-axis generates a two-dimensional sinogram called the “B-scan”, and scanning along the ss-axis generates a stack of B-scans making up a three-dimensional sinogram.

Assuming an acquisition step size of ∆ and an ROI spanning l_fs_ along the fs-axis, acquisition speed along the fs-axis is v_fs_ = PRR ∆ and along the ss-axis is v_ss_ = v_fs_ ∆/l_fs_. Since the step size is much smaller than the dimensions of the ROI, v_fs_ ≫ v_ss_. Clinical RSOM typically involves v_fs_ = 15 mm/s and v_ss_ = 0.045 mm/s. These speeds are such that patient motion can alter the axial position of the transducer relative to the ROI between successive B-scans (Fig. 1b). Consequently, two-dimensional sinograms along the ss-axis appear discontinuous and rippled (Fig. 1d), whereas cross-sections along the fs-axis appear continuous and smooth (Fig. 1c). Such changes in transducer height above the ROI are not taken into account by RSOM reconstruction algorithms published to date^1, 3, 4, 14, 17^.

### The motion correction algorithm

The algorithm is based on the observation that the ultrasound wave front generated by the melanin-containing layers of the skin (primarily the basal layer of the epidermis and the *stratum corneum*) show vertical disruptions in the three-dimensional sinogram. According to the Huygens-Fresnel principle, an ultrasound wave emitted from a continuous surface will generate a secondary wave front with a continuous footprint in the three-dimensional sinogram. Movement during RSOM measurement distorts this footprint and generates discontinuities in the three-dimensional sinogram (Fig. 1d). The motion correction algorithm described here detects disruptions of the *stratum corneum*/*stratum basale* (hereafter “skin surface”) and corrects for this motion by creating an artificial continuous surface. The primary assumption in this algorithm is that the skin surface is continuous on a mesoscopic scale, i.e. on the order of several micrometers.

The motion correction algorithm proceeds in two steps. First, an algorithm is applied to detect the discontinuous skin surface in the three-dimensional sinogram. Second, an artificial, smooth, continuous skin surface is generated based on the detected surface, and the difference between the two surfaces is taken to indicate the vertical movement of the skin with respect to the detector surface. Based on this vertical movement, the focal point of the detector is adjusted during image reconstruction.

**Figure 2:**
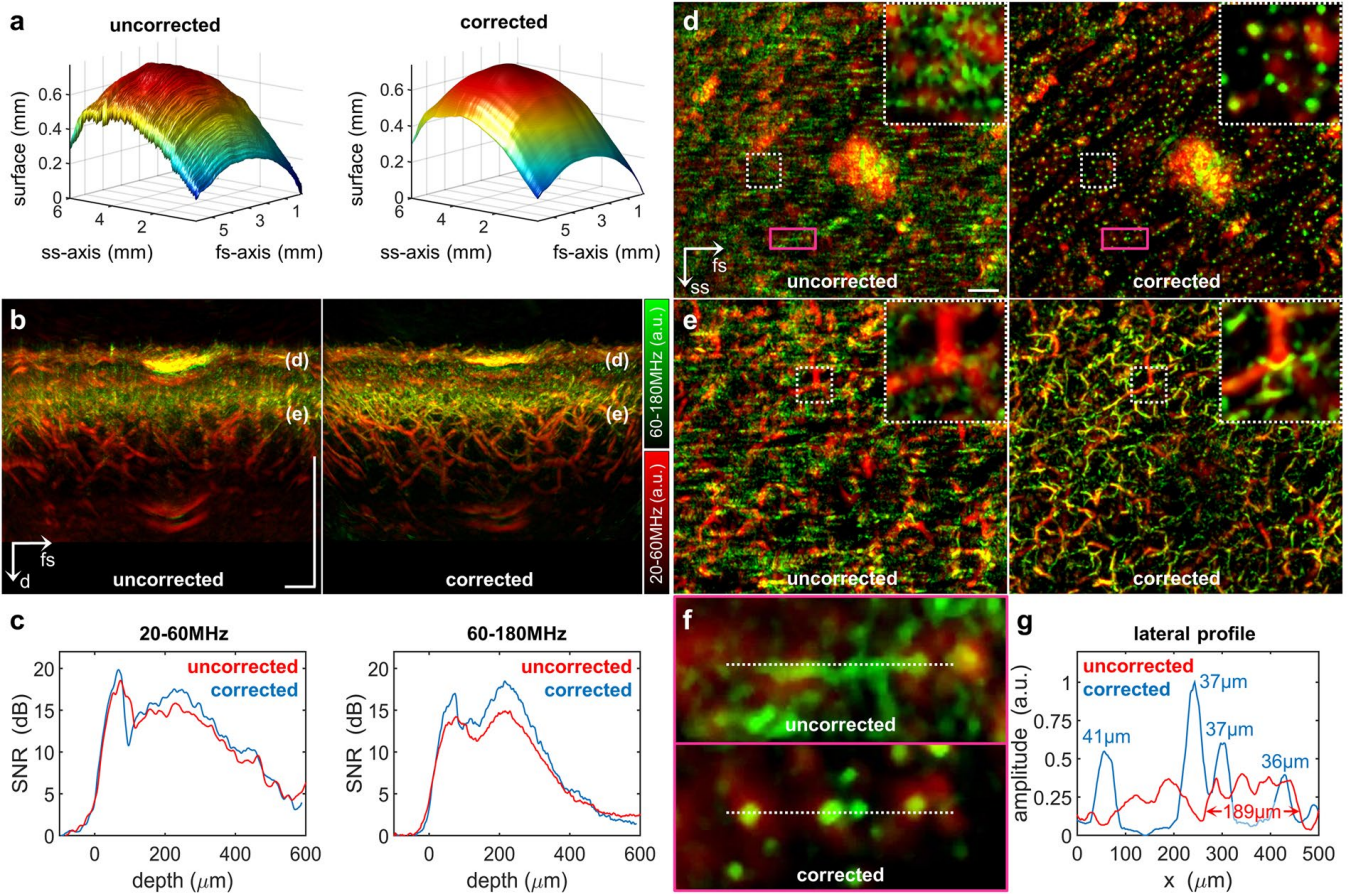
Implementation of the motion correction algorithm in RSOM100. **(a)** Skin surface detected in the raw data before and after motion correction. **(b)** Vertical MAPs of a region of interest on the lower arm surrounding a benign nevus, before and after motion correction. The labels “(**d**)” and “(**e**)” mark the location of the epidermal-dermal junction and the dermal layer shown as lateral MAPs in panels d and e respectively. **(c)** Mean CNR in the cross-sectional MAPs in panel b as a function of depth before and after motion correction. Reconstructions were based on the frequency bands 20–60 MHz (left) or 60–180 MHz (right). **(d)** Lateral MAP of the epidermal-dermal junction before and after motion correction. The insets are zoomed-in views of the areas enclosed in white dashed boxes, showing single capillary loops after motion correction. Magenta boxes enclose areas shown as zoomed-in views in panel f. **(e)** Lateral MAP of an extensive vascular network in the dermal layer, showing well-resolved microvessels (green) after motion correction. **(f)** Zoomed-in views of the regions boxed in magenta in panel d. White dashed lines trace the lateral profile in panel g. **(g)** Lateral profile through four distinct capillary loops. Peak FWHM values are shown. Scale bar, 500 µm. Abbreviations: d: depth; fs: fast-scanning; ss, slow-scanning.

The first step of the algorithm, which is the more challenging, involves detecting the skin surface in the three-dimensional sinogram. We have developed two approaches depending on the tissue being examined. When the ROI lies in an area of hairless skin (e.g. palm of the hand), the *stratum corneum* is roughly segmented using a two-dimensional parabolic slab, reflecting the fact that optoacoustic signal from the *stratum corneum* is displaced by several hundred microns from the signal emitted from underlying vasculature (Fig. 1e). Within the segmented slab, the surface is identified based on the maximum value within each A-scan. When the ROI lies in an area of hairy skin (e.g. lower arm), we use a different approach because the *stratum corneum* is not sufficiently separated in space from the underlying *stratum basale* and dermal papillae (Fig. 1f). Nonetheless, the basal layer of the epidermis leaves a distinctive footprint in the three-dimensional sinogram: ultrasound signals emitted by the *stratum basale* induce shear waves within the acoustic lens of the detector, which in turn stimulate lateral modes in the piezo element of the detector, generating a characteristic low-frequency footprint approximately 600 µm below the skin surface (Fig. 1g). Normally, this low-frequency band is filtered out before reconstruction^1, 3, 4, 14, 17^, but our algorithm exploits it to generate an image of the skin surface. An exponential bandpass filter from 1–3 MHz is applied to the raw data, which are roughly segmented by time to identify the interval containing the highest peak (Fig. 1f). The maximum in the low-frequency band is determined for each A-scan, resulting in a two-dimensional map of the motion-corrupted melanin layer (Fig. 2a).

The second step in the algorithm is the same regardless of whether the skin ROI is hairy or hairless. A moving average filter is applied to the disrupted surface, correcting the entire three-dimensional sinogram and creating an artificially smoothed surface (Fig. 2b). In our experiments, the moving average filter performed best when it spanned 200 pixels along the ss-axis and 10 pixels along the fs-axis. Therefore these span dimensions were used in all experiments described below. The two-dimensional map between the motion-corrupted and artificially smoothed surfaces was calculated to provide the correction for detector z-position during reconstruction.

### *In vivo* validation of the correction algorithm

The performance of the motion correction algorithm was assessed using *in vivo* data collected with three RSOM set-ups based on a spherically focused ultrasound detector with acoustic lens. Human patient procedures were approved by the Ethics Committee of the Technical University of Munich, and all patients provided informed consent. All measurements were performed in compliance with laser safety standards for single laser pulses, pulse trains, and the average power^9^. A derivation of formulas defining maximum exposure limits in optoacoustic mesoscopy have been derived recently^8^.

The high-frequency RSOM set-up (hereafter “RSOM100”) was used to image small vessels in a benign nevus on the lower arm of a healthy man with skin type II. The detector had a detection band of 20–180 MHz, an active aperture of 1.5 mm and an f-number of 1.1. To ensure fast, high-resolution scans, a single-wavelength laser emitting pulses of 532-nm light lasting <1 ns at repetition rates up to 2 kHz was used (Wedge HB532, Bright Solutions SRL, Italy).

A clinical RSOM set-up (hereafter “RSOM55”), in which the scan head was attached to a portable clinical imaging platform^5^, was used to image healthy skin as well as a psoriasis plaque on the arm of an individual diagnosed with psoriasis by physicians at the Klinikum rechts der Isar/Technical University of Munich. The plaque was located on the long head of the triceps brachii, approximately 5 cm above the olecranon. The healthy skin ROI was also located on the long head of the triceps brachii, approximately 4 cm away from the psoriasis plaque. The RSOM55 detector had a detection band of 14–126 MHz, an active aperture of 3 mm, and an f-number of 1. The laser was the same as in the RSOM100 set-up.

During image reconstruction of data collected with the RSOM100 or RSOM55 set-ups, the reconstruction band was split up into low- and high-frequency sub-bands, which were 20-60 and 60-180 MHz for RSOM100 or 14–42 and 42–126 MHz for RSOM55. The two bands were equalized, colored green (high-frequency) or red (low-frequency), and overlaid in final images. Reconstruction using frequency sub-bands improves visualization of higher frequency signals emitted by smaller structures in the tissue^1, 5, 17^.

The third experimental set-up, multispectral RSOM (hereafter “MSOM”), was used to measure blood oxygenation in the lower arm of a healthy man with skin type III. The detector was the same as in the RSOM55 set-up but with a detection band of 14–126 MHz. Tissue was excited using a tunable-wavelength OPO laser emitting 6-ns pulses in the spectral range from 420 to 2300 nm at a pulse repetition rate of 100 Hz (SpitLight OPO, InnoLas Laser GmbH, Krailling, Germany). Other aspects of MSOM imaging, reconstruction, and unmixing were described in a previous publication^6^.

### Parameters used to evaluate the correction algorithm

Uncorrected and corrected reconstructions obtained using the RSOM100 and RSOM55 set-ups were compared in terms of their contrast-to-noise ratio (CNR) as a function of depth. CNR was defined as

1 $$CNR({z}_{k})=20\cdot {\mathrm{log}}_{10}[{R}_{\max }({z}_{k})/nois{e}_{\max }],$$

where ${R}_{\max }({z}_{k})=\mathop{\max }\limits_{i,j}[R({x}_{i},{y}_{j},{z}_{k})]$ is the maximal amplitude within the image plane at depth *z*_*k*_, and *noise*_max_ is the background signal based on the maximal amplitude of the image above the skin surface. The term $R({x}_{i},{y}_{j},{z}_{k})$ represents the three-dimensional reconstruction, where $i=1,2,3,\mathrm{..}.,{N}_{x}$; $j=1,2,3,\mathrm{..}.,{N}_{y}$; and $k=1,2,3,\mathrm{..}.,{N}_{z}$; and *N*_*x*_, *N*_*y*_, and *N*_*z*_ are the numbers of voxels along the x-, y-, and z-axes. The plot of $CNR({z}_{k})$ as a function of depth should show smaller peaks for motion-corrupted data than for data appropriately motion-corrected.

We also compared uncorrected and corrected RSOM100 images in terms of effective resolution calculated from the full width at half maximum (FWHM) of optoacoustic peaks along line profiles corresponding to single capillary loops in the skin. The top part of capillary loops appears as a circular absorber in RSOM images^5^.

We compared uncorrected and corrected MSOM images in terms of blood vessel visualization and blood oxygenation measurement. For blood oxygenation analysis, the same vessel was segmented in motion-corrupted and -corrected three-dimensional reconstructions, and blood oxygenation was measured along the vessel. The segmented vessel was oriented primarily along the y-axis. Blood oxygenation at position *y*_*j*′_ was averaged across the diameter of the vessel using the equation

2 $$s{O}_{2}({y}_{j\text{'}})=\frac{{\sum }_{{i}^{\text{'}},{k}^{^{\prime} }}Seg({x}_{i\text{'}},{y}_{j\text{'}},{z}_{k\text{'}})\cdot s{O}_{2}({x}_{i\text{'}},{y}_{j\text{'}},{z}_{k\text{'}})}{{\sum }_{{i}^{\text{'}},{k}^{^{\prime} }}Seg({x}_{i\text{'}},{y}_{j\text{'}},{z}_{k\text{'}})},$$

where $s{O}_{2}({x}_{i\text{'}},{y}_{j\text{'}},{z}_{k\text{'}})$ is the voxel-to-voxel ratio of oxygenated hemoglobin to total hemoglobin (oxyhemoglobin plus deoxyhemoglobin). The vessel was segmented by thresholding the reconstructed images at 20% of the maximal value:

3 $$Seg({x}_{i\text{'}},{y}_{j\text{'}},{z}_{k\text{'}})=H(R({x}_{i\text{'}},{y}_{j\text{'}},{z}_{k\text{'}})-0.2\cdot \mathop{\max }\limits_{i\text{'},j\text{'},k\text{'}}[R({x}_{i\text{'}},{y}_{j\text{'}},{z}_{k\text{'}})]),$$

where *H*(*x*) is the Heaviside step function and $R({x}_{i\text{'}},{y}_{j\text{'}},{z}_{k\text{'}})$ describes a cuboid in the reconstructed volume that contains the vessel of interest. $Seg(x)=1$ if voxel amplitude is greater than 20% of the maximum amplitude within the cuboid; otherwise, $Seg(x)=0$. Blood oxygenation level $s{O}_{2}({y}_{j\text{'}})$ along the length of blood vessels was compared before and after motion correction. Motion during MSOM data acquisition should give rise to abrupt or large fluctuations in $s{O}_{2}$ that do not overlap with vessel bifurcations.

### Data availability

The datasets generated during and/or analyzed during the current study are available from the corresponding author on reasonable request and with permission of Technical University of Munich and the Helmholtz Zentrum München.

## Results

### Motion correction in high-resolution RSOM100

Figure 2 illustrates the performance of the motion correction algorithm when imaging a benign nevus on the lower arm of a healthy volunteer using the RSOM100 set-up. The algorithm identified a rippled surface in the raw data, indicating micromotion in the z-direction on the order of several micrometers between successive B-scans (Fig. 2a). The skin surface appeared disrupted only along the ss-axis, not along the fs-axis. The motion-corrected skin surface was continuous and smooth, with no offset between neighboring B-scans. Figure 2b displays the vertical maximum amplitude projections (MAPs) of motion-corrupted and motion-corrected imaging volumes. Blurring was visible in the uncorrected image, especially in the high-frequency (green) reconstruction. Figure 2c plots mean CNR with respect to depth based on the vertical MAPs. Motion correction led to higher CNR in the *stratum corneum* as well as superficial horizontal plexus; the CNR increase over uncorrected data was greater at high frequencies (up to 5 dB) than at low ones (up to 2.5 dB). In addition to this quantitative increase in CNR, motion correction led to significant motion artifact removal in the epidermal-dermal junction and the dermis in lateral MAPs (Fig. 2d,e). The increase in effective resolution was particularly apparent at the epidermal-dermal junction (Fig. 2d): the uncorrected MAP showed an unstructured pattern, while the motion-corrected MAP showed a structured pattern of capillary loops. The zoomed-in region (500 µm × 500 µm) in Fig. 2d revealed no individual capillary loops before motion correction, whereas more than a dozen loops were visible after correction. Similarly, microvessels in the papillary dermis were resolved only after motion correction. Inset images in Fig. 2e suggest that micromotion affected small vessels much more than large ones.

To quantify the improvement in effective resolution associated with the motion correction algorithm, line profiles were traced through neighboring capillary loops and analyzed (Fig. 2f,g). While the uncorrected image showed several neighboring capillaries as a lateral tube without clear peaks in the line profile, the corrected image showed four distinctive capillary loops, each appearing as a resolved peak with FWHM values of 36–41 µm. Peaks 3 and 4 combined to form a plateau with FWHM of 189 µm. A walk-through of the motion-corrected three-dimensional volume of the ROI illustrates high resolution of vascular networks at various depths (Supplementary Movie S1).

### Motion correction with clinical RSOM55

**Figure 3:**
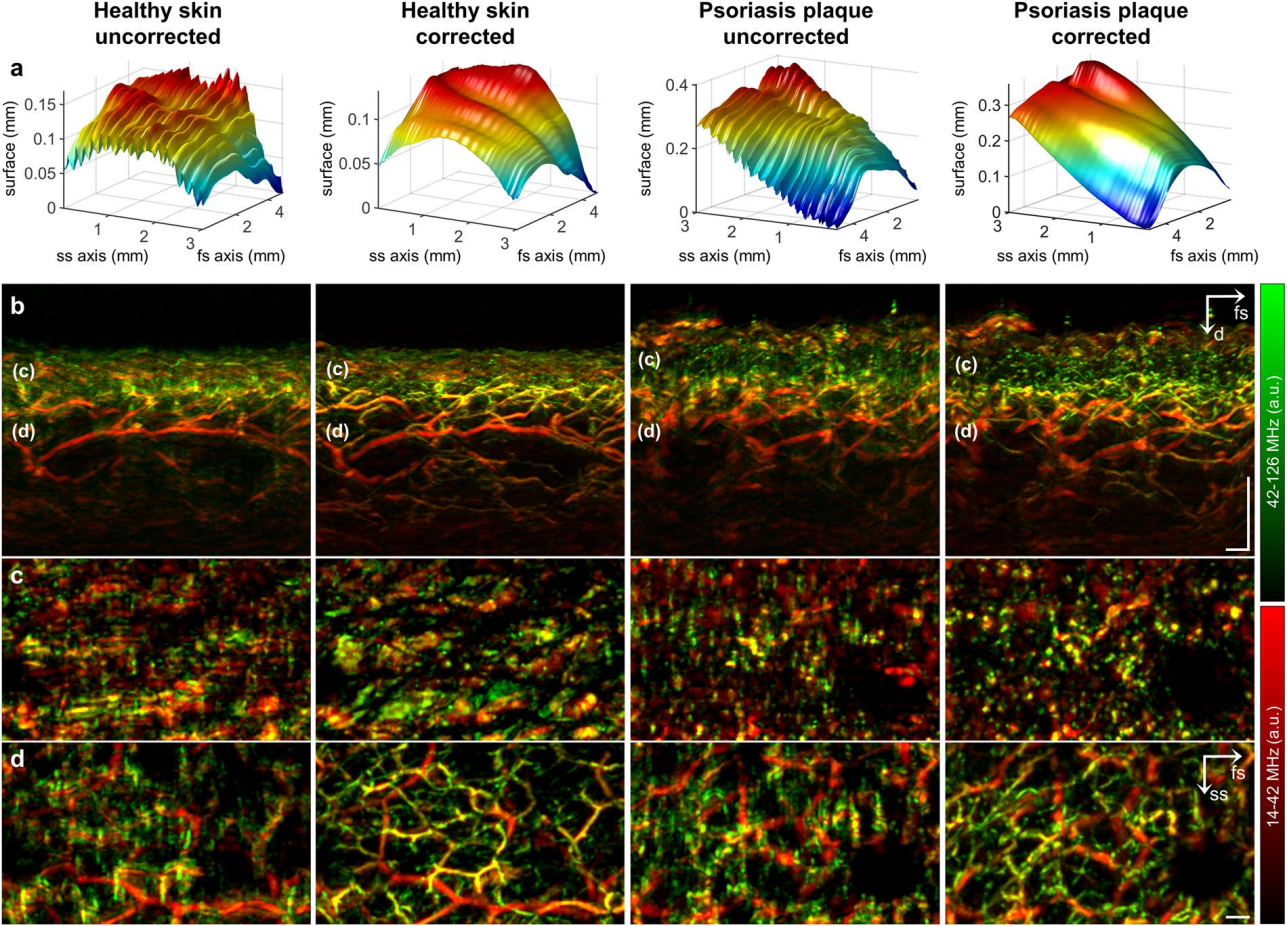
Implementation of the motion correction algorithm in clinical RSOM55. **(a)** Skin surface detected in the raw data before and after motion correction. Periodic disruptions due to breathing are visible in the uncorrected data. **(b)** Vertical MAPs before and after motion correction. Psoriatic skin shows typical thickening of epidermis, broadening of capillary loops, and increased blood volume fraction. The labels “(**c**)” and “(**d**)” mark the location of the epidermal-dermal junction and the dermal layer shown as lateral MAPs in panels c and d respectively (**c**) Lateral MAP of the epidermal-dermal junction. Broadening of capillary loops in psoriatic skin is visible after motion correction. Capillaries in healthy skin are too small to be captured by RSOM55. (**d**) Lateral MAP of an extensive vascular network in the dermal layer, showing well-resolved microvessels (green) only after motion correction. Scale bar, 250 µm. Abbreviations: d: depth; fs: fast-scanning; ss, slow-scanning.

Figure 3a reveals sinusoidal modulation of healthy and psoriatic skin surfaces at approximately 15 cycles per minute (17–18 cycles per measurement), corresponding to the patient’s breathing. This sinusoidal movement was eliminated by the motion correction algorithm, allowing the visualization of inflammation biomarkers typical of psoriatic skin, including thickening of the epidermis, broadening of capillary loops within the dermal papillae, and increased blood volume fraction in dermal vasculature (Fig. 3b–d). Motion correction significantly improved image quality: capillary loops in the psoriasis plaque appeared blurred before motion correction and well resolved afterwards (Fig. 3c). Perhaps the greatest improvement in image quality was observed in the microvasculature of the superficial horizontal plexus (Fig. 3d). Images of both healthy and psoriatic skin revealed a structured network of smaller vessels (green) and larger ones (red) running through the dermis. Smaller vessels were completely blurred before motion correction but well resolved afterwards.

### Motion correction with MSOM

**Figure 4:**
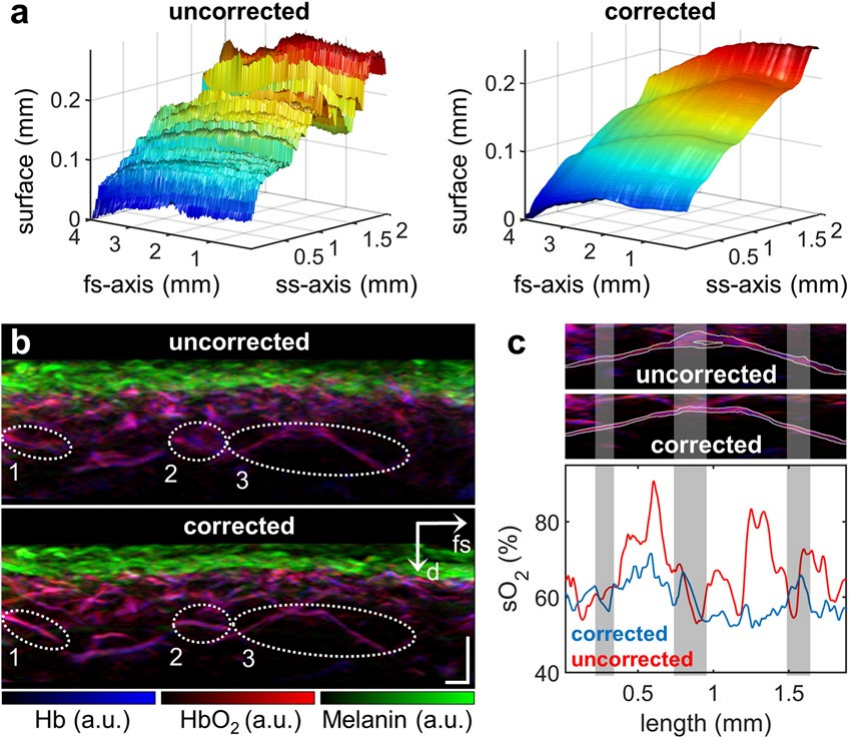
Implementation of the motion correction algorithm in MSOM. **(a)** Skin surface of healthy lower arm area before and after motion correction. Strong motion artifacts are visible even along the fs-axis because of the long measurement time necessary for multispectral acquisition. Nevertheless, the algorithm is able to generate a smooth, continuous surface. **(b)** Vertical MAP of unmixed chromophores in the lower arm. Individual vessels enclosed by ellipses 1 and 2 are resolved only after motion correction. The vessel enclosed by ellipse 3 is further analyzed in panel c. **(c)** Segmentation of the vessel within ellipse 3 in panel b and outlined in white (top and middle panels), followed by measurement of blood oxygenation (oxygen saturation, sO_2_) across the diameter of the blood vessel within the segmented area (lower panel). The oxygenation level is plotted from left to right along the vessel. Gray bands indicate regions of vessel bifurcation. Scale bar, 250 µm. Abbreviations: d: depth; fs: fast-scanning.

By measuring three-dimensional absorption maps at multiple wavelengths, MSOM provides functional information based on the spectral signature of intrinsic chromophores. Figure 4 shows the impact of motion on the visualization of vasculature as well as the quantification of blood oxygenation in the lower arm of a healthy volunteer. Figure 4a shows that without motion correction, the skin surface appeared strongly rippled, even along the fs-axis, reflecting the prolonged measurement time needed for MSOM data acquisition. The motion-corrected surface was smooth and continuous along both the fs- and ss-axes. Figure 4b highlights two areas (ellipses 1 and 2) where single blood vessels appeared as a cloud without clear boundaries prior to motion correction but as well-resolved vessels afterwards. Another vessel (ellipse 3) appeared as a double structure before motion correction, and the “shadow vessel” disappeared after correction (Fig. 4b,c).

In addition to affecting blood vessel visualization in MSOM, motion also interfered with measurement of blood oxygenation. The vessel within ellipse 3 in Fig. 4b was segmented and mean blood oxygenation was measured along its length (Fig. 4c). Blood oxygenation level in the uncorrected image fluctuated sharply between 55% and 90%, and the fluctuations did not correlate with vessel bifurcations (gray bands). Blood oxygenation level in the motion-corrected image showed smaller, less abrupt variations, ranging from 55% to 70%. In addition, regions of vessel bifurcation were associated with changes in blood oxygenation.

## Discussion

In this study, we present to our knowledge the first motion correction algorithm for RSOM, enabling motion artifact removal with effective resolution up to 5-fold better than conventional RSOM. We show that by detecting the disrupted skin surface in raw data and comparing it to an artificially smoothed, continuous surface, we can reliably correct for motion effects during image reconstruction in optoacoustic dermoscopy. We validate the algorithm using high-frequency RSOM100, clinical RSOM55, and functional MSOM set-ups.

The RSOM100 set-up is particularly vulnerable to motion artifacts because it can offer higher resolution of 4 µm axially and 20 µm laterally^1, 17^ at slower acquisition speeds of ~80 pixels per second^8^. We have shown that our motion correction algorithm can provide unparalleled RSOM100 imaging of capillary loops in the epidermal-dermal junction as well as the superficial horizontal plexus.

Clinical RSOM55, although in principal less vulnerable to motion artifacts than RSOM100 because it images at lower resolutions of 10 µm axially and 40 µm laterally and faster acquisition speeds of ~140 pixels per second^5, 17^, must perform under challenging conditions. Various parts of the body, some difficult to hold still, must be imaged in the clinic, including lower extremities, trunk, elbow and scalp^25^, and most individuals being scanned are elderly or unwell and may have difficulty controlling their breathing or trembling. Imaging under these conditions is more demanding than imaging the palm^3, 4, 10, 12, 26^ or lower arm^8, 11^ of healthy individuals under laboratory conditions. We have shown that our motion correction algorithm can correct for strong breathing motion enabling quantification of disease biomarkers even under challenging conditions.

Functional MSOM is extremely sensitive to motion because it acquires only 3.4 pixels per second^8^, meaning that a scan of 4 mm × 2 mm takes approximately 13 minutes^6^, at a lateral resolution of approximately 55 µm^27^. Each of the datasets acquired at different wavelengths shows unique motion artifacts in the uncorrected multi-wavelength reconstructions. Thus, not only contrast is affected by motion but the spectral signature of individual image pixels as well. Simple co-registration of the data based on Euclidean transformation is not sufficient to perfectly co-register each pixel in inter-wavelength reconstructions of uncorrected datasets. We have shown that without motion correction, MSOM not only fails to resolve anatomical structures but also generates highly unreliable blood oxygenation measurements.

The motion correction algorithm presented in this work was designed for optoacoustic mesoscopy, which requires tomographic reconstruction. In comparison, optoacoustic microscopy, which does not need 3D reconstruction, will not benefit from the proposed algorithm significantly since motion artifacts are restricted to the axial dimension and do not propagate to the lateral dimension. Yet optoacoustic mesoscopy outperforms optoacoustic microscopy in imaging depth. The imaging depth in optoacoustic microscopy is limited by the high scattering coefficient of human skin, which is approximately twice as strong compared to subcutaneous tissue, which in turn is stronger than muscle tissue^28^. At a wavelength of 532 nm the reduced scattering coefficient of human skin measures approximately µ_s_’ = 50 cm^−1^, severely limiting the possibility to optically focus light beyond a few hundred micron. Favazza *et al*. have performed a direct comparison of optoacoustic micrsocopy and mesocopy and showed that microscopy can resolve only capillaries and small vessels of the upper superficial horizontal plexus, whereas mesoscopy can observe all vasculature within the dermis^29^. We have recently shown that optoacoustic mesoscopy is even capable of measuring all vasculare structures within the dermis from the small capillaries in the epidermal-dermal junction to the larger vessels of the deep horizontal plexus in the dermis^8^.

The algorithm presented here works by identifying the footprint of the skin surface in three-dimensional sinograms, which is facilitated by the strongly absorbing *stratum basale*. In the case of humans or animals lacking an absorbing layer close to the skin surface, such a layer can easily be created by dissolving ink in the acoustic coupling agent, by slightly coloring the skin surface with a marker, or by introducing absorbing molecules in the coupling membrane. This is analogous to marking the skin surface with a fiducial marker in optical coherence tomography^30^. Thus, the motion correction algorithm described here is applicable to a wide range of preclinical and clinical RSOM studies.

This motion correction algorithm will help provide the accuracy and reliability needed for realizing the full clinical potential of RSOM. This technique, already optimized in terms of frequency and excitation energy^3, 4, 8^, has shown the ability to measure angiogenesis in preclinical models of melanoma tumors, and phenotypic biomarkers of inflammation in human skin^5, 17^. The multispectral capability of MSOM can provide readings of dermal blood oxygenation level and melanin content^6^. The algorithm presented in this work will allow for a wide range of interesting RSOM-based dermatology studies in the near future.

## Electronic supplementary material

Movie S1
